# Using routinely collected laboratory data to identify high rifampicin-resistant tuberculosis burden communities in the Western Cape Province, South Africa: A retrospective spatiotemporal analysis

**DOI:** 10.1371/journal.pmed.1002638

**Published:** 2018-08-21

**Authors:** Avery I. McIntosh, Helen E. Jenkins, Laura F. White, Marinus Barnard, Dana R. Thomson, Tania Dolby, John Simpson, Elizabeth M. Streicher, Mary B. Kleinman, Elizabeth J. Ragan, Paul D. van Helden, Megan B. Murray, Robin M. Warren, Karen R. Jacobson

**Affiliations:** 1 Department of Biostatistics, Boston University School of Public Health, Boston, Massachusetts, United States of America; 2 National Health Laboratory Service, Cape Town, South Africa; 3 Department of Global Health and Social Medicine, Harvard Medical School, Boston, Massachusetts, United States of America; 4 DST/NRF Centre of Excellence for Biomedical Tuberculosis Research/SAMRC Centre for Tuberculosis Research, Division of Molecular Biology and Human Genetics, Faculty of Medicine and Health Sciences, Stellenbosch University, Cape Town, South Africa; 5 Section of Infectious Diseases, Boston University School of Medicine and Boston Medical Center, Boston, Massachusetts, United States of America; Centers for Disease Control and Prevention, UNITED STATES

## Abstract

**Background:**

South Africa has the highest tuberculosis incidence globally (781/100,000), with an estimated 4.3% of cases being rifampicin resistant (RR). Control and elimination strategies will require detailed spatial information to understand where drug-resistant tuberculosis exists and why it persists in those communities. We demonstrate a method to enable drug-resistant tuberculosis monitoring by identifying high-burden communities in the Western Cape Province using routinely collected laboratory data.

**Methods and findings:**

We retrospectively identified cases of microbiologically confirmed tuberculosis and RR-tuberculosis from all biological samples submitted for tuberculosis testing (*n* = 2,219,891) to the Western Cape National Health Laboratory Services (NHLS) between January 1, 2008, and June 30, 2013. Because the NHLS database lacks unique patient identifiers, we performed a series of record-linking processes to match specimen records to individual patients. We counted an individual as having a single disease episode if their positive samples came from within two years of each other. Cases were aggregated by clinic location (*n* = 302) to estimate the percentage of tuberculosis cases with rifampicin resistance per clinic. We used inverse distance weighting (IDW) to produce heatmaps of the RR-tuberculosis percentage across the province. Regression was used to estimate annual changes in the RR-tuberculosis percentage by clinic, and estimated average size and direction of change was mapped. We identified 799,779 individuals who had specimens submitted from mappable clinics for testing, of whom 222,735 (27.8%) had microbiologically confirmed tuberculosis. The study population was 43% female, the median age was 36 years (IQR 27–44), and 10,255 (4.6%, 95% CI: 4.6–4.7) cases had documented rifampicin resistance. Among individuals with microbiologically confirmed tuberculosis, 8,947 (4.0%) had more than one disease episode during the study period. The percentage of tuberculosis cases with rifampicin resistance documented among these individuals was 11.4% (95% CI: 10.7–12.0). Overall, the percentage of tuberculosis cases that were RR-tuberculosis was spatially heterogeneous, ranging from 0% to 25% across the province. Our maps reveal significant yearly fluctuations in RR-tuberculosis percentages at several locations. Additionally, the directions of change over time in RR-tuberculosis percentage were not uniform. The main limitation of this study is the lack of unique patient identifiers in the NHLS database, rendering findings to be estimates reliant on the accuracy of the person-matching algorithm.

**Conclusions:**

Our maps reveal striking spatial and temporal heterogeneity in RR-tuberculosis percentages across this province. We demonstrate the potential to monitor RR-tuberculosis spatially and temporally with routinely collected laboratory data, enabling improved resource targeting and more rapid locally appropriate interventions.

## Abstract 2

Using the provincial laboratory database from the Western Cape, South Africa, Karen Jacobson and colleagues demonstrate a method for real-time monitoring of drug-resistant tuberculosis.

## Author summary

**Why was this study done?:**

- The World Health Organization’s End TB strategy sets large goals in the reduction of tuberculosis incidence and mortality; however, past efforts in the control and eradication of infectious disease demonstrate the critical importance of understanding the spatial and temporal distribution of disease.
- Tuberculosis is now the leading cause of death by infectious disease in the world, making it a global health priority.
- South Africa, a country with one of the highest tuberculosis incidence rates globally, has a centrally collected laboratory database, providing the infrastructure to create an urgently needed national surveillance system.

**What did the researchers do and find?:**

- South Africa’s centralized laboratory database lacks unique patient identifiers, necessitating the use of a person-matching algorithm to link repeat and longitudinal specimens to (a) the same individual and (b) to the same episode of disease. We present our methods for linking the provincial laboratory database from the Western Cape, South Africa.
- We linked a total of 2,219,891 samples submitted for tuberculosis testing between 2008 and 2013 to 799,779 individuals who were mappable to clinic locations. A total of 222,735 (27.8%) had microbiologically confirmed tuberculosis, of which 10,255 (4.6%, 95% CI: 4.6–4.7) had documented rifampicin resistance, a marker for multidrug-resistant TB.
- RR-tuberculosis was spatially heterogeneous, ranging from 0% to 25% across the province. Additionally, our maps reveal significant yearly fluctuations in RR-tuberculosis percentages at several locations.

**What do these findings mean?:**

- Through this project, we have developed a road map for organizing routinely collected laboratory data from the South African NHLS, showing that molecular diagnostics can be transformed into a meaningful surveillance tool.
- Our maps reveal striking spatial and temporal heterogeneity in RR-tuberculosis percentages across this province. We demonstrate the potential to monitor RR-tuberculosis spatially and temporally with routinely collected laboratory data, enabling improved resource targeting and more rapid locally appropriate interventions.
- Our reported estimates are approximations, reliant on the accuracy of our person-matching algorithm and adherence to testing algorithms. South Africa would benefit from adoption of unique patient identifiers integrated into the specimen capture systems to support stronger surveillance and tracking.

## Introduction

In 2015, tuberculosis became the leading infectious disease killer globally, responsible for more than 4,500 deaths daily [1]. The World Health Organization (WHO) End Tuberculosis Strategy aims to reduce tuberculosis deaths by 95% and disease incidence by 90% before 2035 [2]. Given the current 1.9% annual decline in tuberculosis incidence [1,2], dramatic changes to the tuberculosis control strategy are required to meet WHO targets [2]. Multidrug-resistant (MDR) tuberculosis, defined as resistance to both rifampicin and isoniazid, is a significant barrier to successful tuberculosis control because of diagnostic delays, high treatment failure rates, and the cost burden on health systems [3,4]. Control and elimination strategies for other diseases, such as polio and smallpox, have only succeeded by using detailed spatial information to understand where and why pathogens persist and then tailoring interventions accordingly [5–9]. To make tuberculosis elimination affordable, sustainable, and effective, the global control program needs surveillance systems to monitor drug resistance and identify high burden communities from regularly collected, routine data [10].

South Africa has one of the highest tuberculosis incidence rates globally (781/100,000) [1], yet relatively little is known about the spatial heterogeneity of tuberculosis or MDR-tuberculosis within the country. A recent modeling study predicts that South African MDR-tuberculosis incidence will double by 2040 [11]. In contrast, the 2012–2014 South African Tuberculosis Drug Resistance Survey showed no change in national MDR-tuberculosis prevalence (2.8%, 95% CI: 2.0%–3.6%) compared to the previous 2001–2002 survey [12]. In the Western Cape Province, the 2012–2014 survey reports an MDR-tuberculosis prevalence of 3.0% (95% CI: 2.1%–4.2%) and 4.3% (95% CI: 3.2%–5.5%) for any rifampicin resistance [12]. The spatial distribution of cases was not reported below the provincial level. Drug resistance surveys, although important for public health planning, are logistically complex, expensive, and limited in usefulness for identifying transmission hot spots because of lack of spatial granularity and delay between data acquisition and final reporting.

Investigators in other settings have derived spatial estimates of drug-resistant tuberculosis and monitored outbreaks by aggregating and analyzing WHO data, leveraging country-specific surveillance systems, prospectively collecting cohort data, or using social network analysis [13–16]. In a country such as South Africa, with a high tuberculosis disease burden and a centrally collected laboratory database, the infrastructure exists to create an urgently needed surveillance system. The South African laboratory database became more suited for surveillance purposes after the introduction of new testing algorithms using rapid tuberculosis molecular tests, such as the Genotype MTBDR*plus* line probe assay (LPA; Hain Lifescience GmbH, Nehren, Germany) in 2008 and Xpert MTB/RIF (Xpert; Cepheid, Sunnyvale, CA) in 2011. These algorithms include performing drug susceptibility tests (DSTs) for rifampicin on all individuals with microbiologically confirmed tuberculosis, either sequentially or simultaneously, and collecting those results in a centralized database [17]. However, patients are not reliably assigned unique identifiers in this system, and each patient may have multiple specimens. Without unique identifiers, specimens remain unlinked, thus hindering the ability to aggregate unique cases spatially. This deficit necessitates the development of methods to organize specimen results spatially at the individual-person level. In this paper, we describe a method to link routinely collected laboratory data from the Western Cape Province National Health Laboratory Services (NHLS) to create unique patient identifiers. We then map the spatial distribution of microbiologically confirmed rifampicin-resistant (RR) tuberculosis cases, which is the diagnosis used to initiate MDR treatment, in order to identify areas of high disease burden and to monitor changes over time.

## Methods

### Patient population and data source

We performed a retrospective study of all tuberculosis tests submitted to the Western Cape Province NHLS between January 1, 2008, and June 30, 2013. The 2011 South African Census reports the Western Cape Province population as 5,822,734, dominated by the Cape Town metropole, with 3,740,026 residents [18]. With a few exceptions, all samples from persons being evaluated for tuberculosis in the public sector in the Western Cape are sent to NHLS for diagnosis. From 2008 to 2011, all patients with a positive smear and/or culture for *Mycobacterium tuberculosis* were to reflexively receive LPA to test for isoniazid and rifampicin resistance [19]. Since the introduction of Xpert in 2011 [20], two samples are routinely sent to NHLS: one for Xpert and, if Xpert indicates rifampicin-susceptible *M*. *tuberculosis* complex, one for acid-fast bacilli (AFB) smear. If rifampicin resistance is detected on Xpert, the second sample is subjected to decontamination, smear microscopy, mycobacterial culture, and resistance results are confirmed by LPA.

Because the NHLS sample database lacks reliable unique patient identifiers, we created a person-matching algorithm using name, surname, age, location, and sex to match and link specimen records to individual patients. Specimens with no name or location were excluded. Specimens that had the same patient name with a recorded age within two years of one another and had either the same physical location of sample collection or patient sex were designated as coming from the same individual. We ran specimens labeled with common surnames through a stricter algorithm requiring full matches on all aforementioned fields, as detailed in the supporting information (S1 Text). If an individual had an additional positive tuberculosis test within a year of the first, we removed the later result to avoid multiple positive tests for the same disease episode (further detailed in S2 Text). The same individual was defined as having multiple disease episodes if they had positive tests for tuberculosis greater than two years apart within our study window. We defined more than two years as being a new episode to reflect the increased likelihood at that point of being reinfection rather than ongoing treatment, treatment failure, or relapse [21].

We abstracted AFB sputum grade, mycobacterial culture result, rifampicin and isoniazid LPA resistance results, sample collection location, and test result date. Because Xpert was used only midway during this study period, and we did not have access to all Xpert results, we did not include Xpert results as part of a case definition. We defined a case of microbiologically confirmed tuberculosis as having a positive AFB smear or mycobacterial culture. We considered individuals with no microbiologic evidence of tuberculosis and at least one negative culture result to have a negative test. Individuals with only indeterminate tests, which included empty or insufficient samples submitted or contaminated samples, were considered indeterminate. We designated a patient with rifampicin resistance on LPA or phenotypic DST result on culture as a RR-tuberculosis case because these are the confirmatory tests in the NHLS system (details in S3 Text) [3]. Individuals with documented rifampicin susceptibility on LPA or phenotypic DST, and those for whom DST were not available in our dataset, were considered to have rifampicin-susceptible tuberculosis disease.

If a specimen that was positive for tuberculosis and was submitted from a clinic, we assigned the tuberculosis episode for that specimen to that clinic location. Individuals with positive specimens submitted from two different clinics within two years of each other were assigned to the first clinic from which a specimen was submitted. If an individual tests RR any time during their tuberculosis episode, then that episode is a RR-tuberculosis episode and assigned to the initial clinic location. We removed individuals with samples submitted exclusively from non-clinic locations (military, correctional, or psychiatric facilities, women’s shelters, or hospitals) on the assumption that cases from non-clinic locations originate from a broader community and therefore do not reflect the spatial distribution of disease in the location where they were tested. Individuals diagnosed at a non-clinic location with an additional specimen submitted from a clinic at any point were assigned to the clinic closest in time to their positive specimen, based on the assumption that the clinic location reflected his/her area of residence. We removed smaller clinics with fewer than 20 tuberculosis episodes over the study period to avoid the introduction of clinics with very small sample sizes and therefore very low precision. These smaller clinics also do not regularly evaluate individuals for tuberculosis, reflected by their small number of samples submitted.

The study was approved by Stellenbosch University’s Health Research Ethics Committee and the Boston Medical Center’s Institutional Review Board. Given the retrospective nature of the study, a waiver for informed consent was granted.

### Mapping and statistical analyses

The Western Cape Province is divided administratively into 6 districts and 25 subdistricts. We mapped each clinic’s geo-coordinates and obtained district and subdistrict population estimates from the most recent 2011 South African population census [18]. We used ArcGIS version 10.3 [22] to generate maps of clinic locations, subdistrict populations, aggregated tuberculosis and RR-tuberculosis case counts at the subdistrict level, and heatmaps for visualization of interpolated rates of RR-tuberculosis as a percentage of tuberculosis diagnosed between clinic locations.

Case counts of tuberculosis and RR-tuberculosis were aggregated at the clinic level for each year and over the entire study period. A percentage of RR-tuberculosis cases for all tuberculosis cases was calculated for each clinic. We used a standard inverse distance weighting (IDW) technique implemented in ArcGIS to pixelate the Western Cape and generate a color-coded interpolated RR-tuberculosis percentage for each unobserved pixel, further detailed in S4 Text. By using a standard IDW approach, we assume that things that are close to one another are more alike than those that are far apart, reflecting that individuals attend a clinic closest to their residence to receive a tuberculosis diagnosis and, therefore, that the clinic represents the more proximal area.

To visualize the evidence for changing RR-tuberculosis percentages over time in a single map, we estimate the change in the percentage of RR-tuberculosis via a regression model with year as the predictor and the yearly number of RR-tuberculosis cases as the outcome, with an offset term of the total number of tuberculosis cases diagnosed that year at the clinic. We fit a Poisson regression at each clinic unless a clinic location had less than four years of data, in which case we fit a negative binomial regression for better estimability. We mapped the results of these clinic-level models by plotting one minus the *p*-value estimating the statistical significance of the annual change in RR-tuberculosis percentages, and then making the value negative if the model estimated that there was a decrease in RR-tuberculosis over time. For example, a clinic with an estimated decrease in RR-tuberculosis over time and a *p*-value for that trend of 0.05 would be assigned −0.95 for the mapping exercise, while a clinic with similar statistical significance but an increasing RR-tuberculosis percentage through time would be assigned 0.95 for mapping. This transformation restricts the range of transformed *p*-values between −1 and 1, and provides a measure of evidence for the likelihood that the direction of change of the RR rate, either positive or negative over time, is not due to inherent variability in the percentage. These analyses are intended to identify locations that warrant further investigation. All statistical calculations were performed using the R statistical software package version 3.3.1 [23]. Data are deposited in the Dryad repository, https://doi.org/10.5061/dryad.34hp52d [24].

## Results

**Fig 1:**
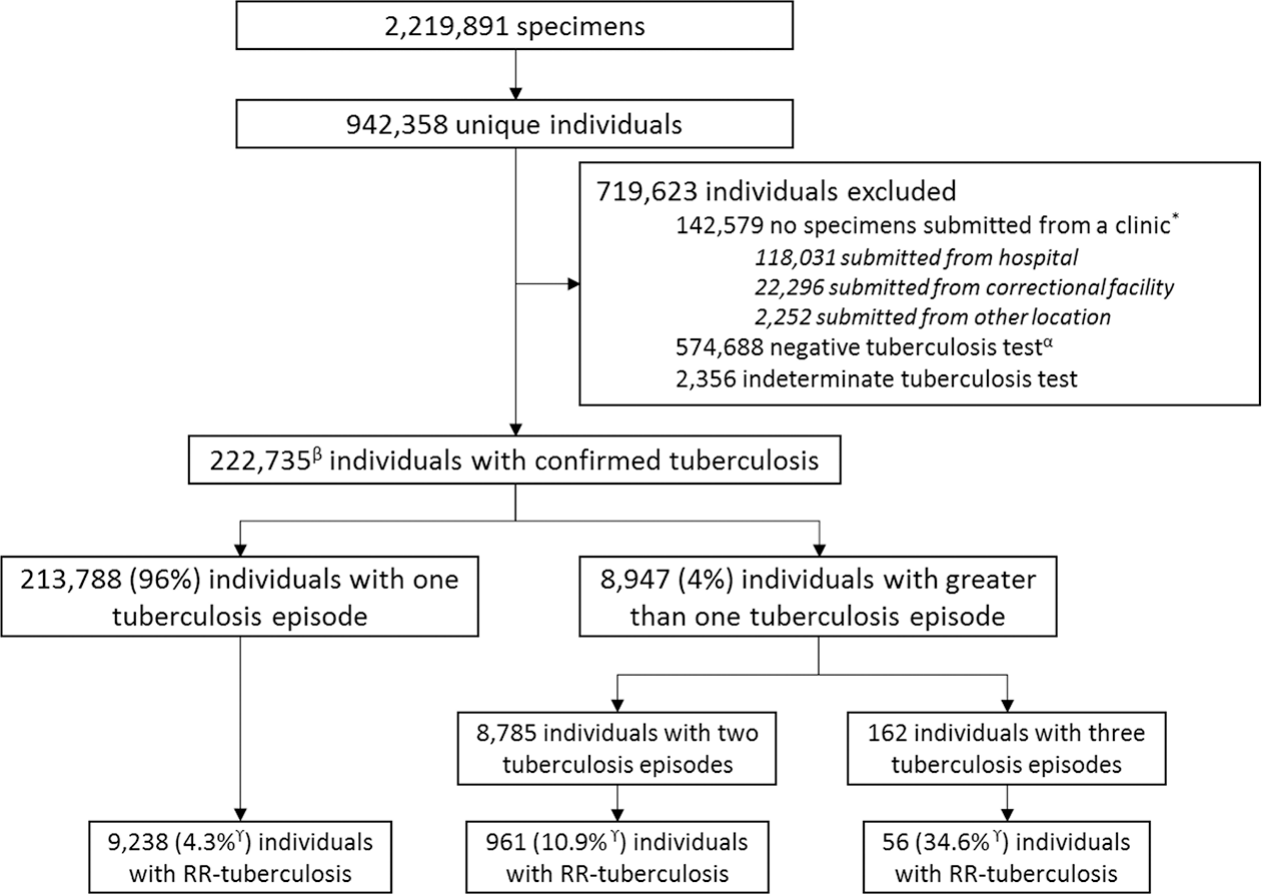
Flow diagram showing initial specimens from NHLS, person-linkage results in unique individuals, reasons for exclusion from final mapping, and final results stratified by number of tuberculosis episodes and number of individuals with RR-tuberculosis. *Locations of individuals with no specimens submitted from a clinic, indicating the location of the first specimen submitted for a given individual. ^α^Individuals without a positive tuberculosis test and at least one negative tuberculosis test. ^β^Of these individuals with microbiologic confirmation of tuberculosis, 109,246 (49%) had documented rifampicin DST results, either Genotype MTBDR*plus* LPA or culture based. ^γ^The percentage of individuals with RR-tuberculosis is calculated as the number of RR-tuberculosis cases diagnosed by DST divided by all individuals with microbiologically confirmed tuberculosis. Because a proportion did not complete the rifampicin resistance testing algorithm, this percentage is a lower boundary. DST, drug susceptibility test; LPA, line probe assay; NHLS, National Health Laboratory Services; RR, rifampicin-resistant.

Between January 1, 2008, and June 30, 2013, 2,219,891 biological specimens, of which 94% were sputum and bronchial, were submitted for tuberculosis testing to the NHLS; these specimens had a person-name and location from which they were submitted. We removed 47,147 samples (2.1%) because of lack of person-name or location. After applying the person-matching algorithm, we counted 942,358 unique individuals in the database (Fig 1). Of these individuals, 799,779 (85%) were mappable to a clinic location: 748,691 (80%) had specimens submitted for testing solely from a clinic and 51,088 (5%) had tests submitted from a clinic and at least one additional location. The remaining 142,579 (15%) individuals had tests submitted exclusively from non-clinic locations and were therefore excluded from further analyses (S1 Table details the non-clinic locations from which samples were submitted). The percentage of individuals with microbiologically confirmed tuberculosis did not differ between clinic and non-clinic locations (27.8% versus 28.0%, *p* = 0.16). The percentage of microbiologically confirmed RR-tuberculosis was lower for individuals with at least one specimen submitted from a clinic compared to individuals with specimens submitted only from non-clinic locations (4.6% versus 6.7%, *p* < 0.0001).

Of the 799,779 individuals with tests submitted from clinics, 222,735 (27.8%) had microbiologically confirmed tuberculosis. Of those with microbiologically confirmed tuberculosis, 109,246 (49%) had rifampicin susceptibility documented by LPA or culture-based techniques; the 51% without documented results were assumed to have rifampicin-susceptible tuberculosis. Accounting for multiple tuberculosis episodes from the same person, 231,844 distinct tuberculosis episodes were identified. The individuals with microbiologically confirmed tuberculosis were 43% female and had a median age of 36 years (IQR: 27–44). Of the 231,844 tuberculosis episodes, 10,762 (4.6%, 95% CI: 4.5–4.7) were RR-tuberculosis and 8,205 episodes (3.5%, 95% CI: 3.5–3.6) had both rifampicin and isoniazid resistance documented (Table 1). Among individuals with microbiologically confirmed tuberculosis, 8,947 (4.0%) had more than one episode of disease during the study period. Among individuals with more than one episode, the percentage with RR-tuberculosis was 11.4% (95% CI: 10.7–12.0).

**Table 1:** Number of tuberculosis episodes mappable to clinics by year, and the breakdown of tuberculosis episodes among individuals with only one episode or with more than one episode of tuberculosis during the study period, and the number of RR-tuberculosis episodes each year, with percentages of the total number of tuberculosis episodes for the given year.

Number of episodes	2008	2009	2010	2011	2012	2013*	Total
One episode (%)^≠^	45,809 (92.0%)	40,867 (92.4%)	41,276 (92.4%)	36,039 (92.5%)	29,493 (92.7%)	20,304 (90.9%)	213,788 (92.2%)
≥One episode (%)^α^	3,989 (8.0%)	3,383 (7.6%)	3,411 (7.6%)	2,912 (7.5%)	2,323 (7.3%)	2,038 (9.1%)	18,056 (7.8%)
RR-tuberculosis (%)º	2,304 (4.6%)	1,766 (4.0%)	1,734 (3.9%)	1,702 (4.4%)	1,752 (5.5%)	1,504 (6.7%)	10,762 (4.6%)
Total episodes	49,798	44,250	44,687	38,951	31,816	22,342	231,844

*Reflects a partial year, January through June 2013.

^≠^Episodes of tuberculosis among individuals who were not found to have any additional episode of tuberculosis during the study period.

^α^Episodes of tuberculosis among individuals who were found to have an additional episode of tuberculosis during the study period.

ºEpisodes of RR-tuberculosis among all episodes of disease.

Abbreviation: RR, rifampicin-resistant.

**Fig 2:**
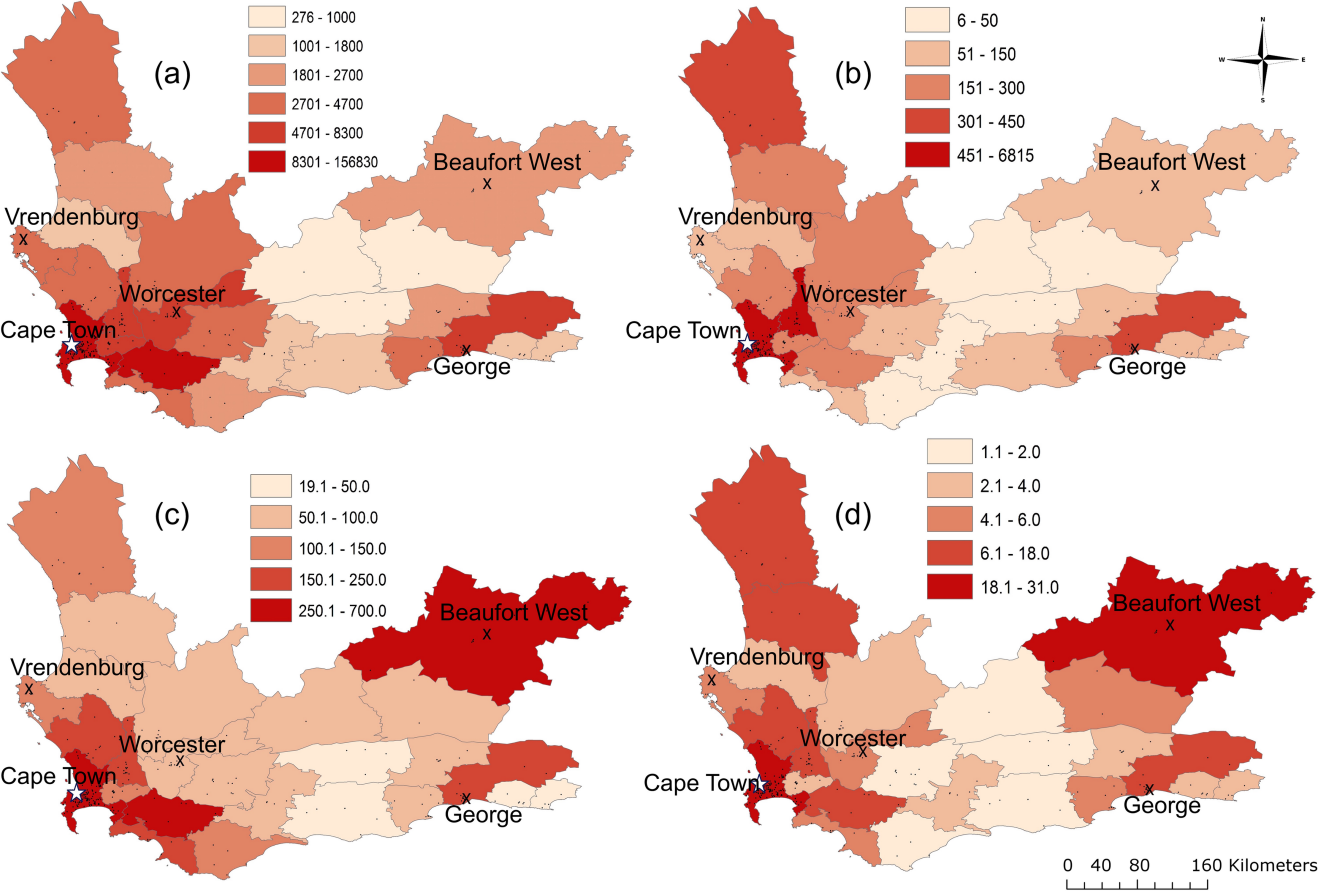
Tuberculosis case counts and RR-tuberculosis case counts aggregated by subdistrict (a and b) and per 100,000 population (c and d). (a) Clinic-diagnosed tuberculosis case counts at subdistrict level between January 2008 and June 2013. (b) Clinic-diagnosed RR-tuberculosis case counts at subdistrict level for the same period. (c) Clinic-diagnosed tuberculosis case count at subdistrict per 100,000 population per year. (d) Clinic-diagnosed RR-tuberculosis case count per 100,000 population per year. Black points denote clinic locations, X’s denote large towns, and the star denotes the Cape Town metropole. RR, rifampicin-resistant.

To discern geographic differences in disease distribution, we aggregated mappable tuberculosis and RR-tuberculosis case counts at the subdistrict level (Fig 2A and 2B). The highest case counts were in subdistricts containing the largest metropolitan areas (Cape Town and George). However, subdistricts with the highest RR-tuberculosis case counts did not necessarily have the highest tuberculosis case counts; some regions with a high number of RR-tuberculosis cases had a low number of tuberculosis cases. This discrepancy became more apparent after adjusting for population (Fig 2C for tuberculosis and 2d for RR-tuberculosis). For example, the subdistricts of Matzikama and Cederberg in the West Coast had among the highest mappable RR-tuberculosis counts (18 and 11 per 100,000 population) but not the highest mappable tuberculosis counts (144 and 95 per 100,000 population). Conversely, Overstrand and Cape Agulhas subdistricts in the south had relatively high tuberculosis counts (247 and 119 per 100,000 population) but low RR-tuberculosis counts (5 and 2 per 100,000 population).

**Fig 3:**
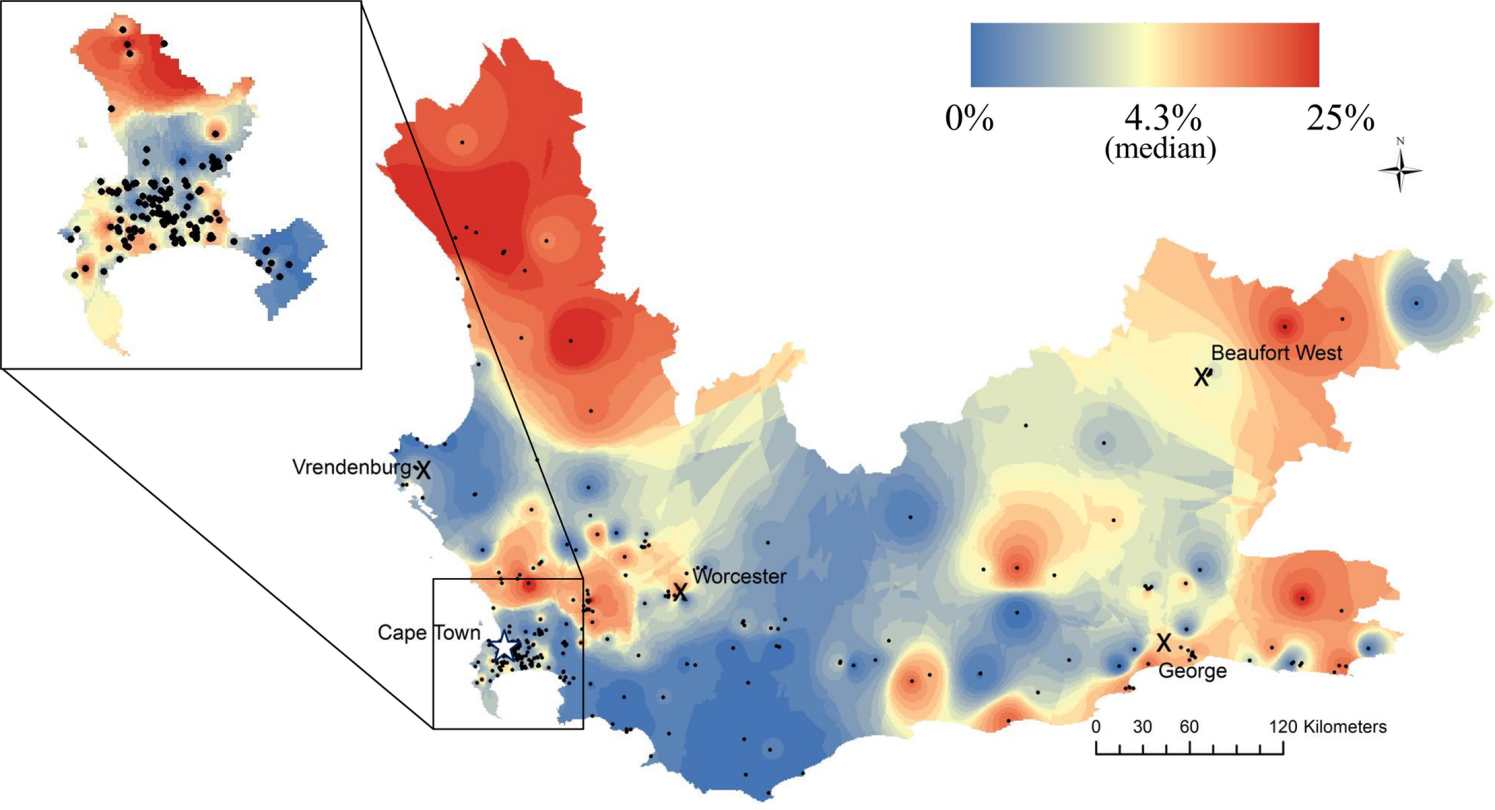
The percentage of total clinic-diagnosed tuberculosis cases found to be rifampicin resistant in Western Cape Province between 2008 and 2013. Colors are broken by quantiles. Black dots denote clinic locations, X’s denote large towns, and the star denotes the Cape Town metropole. Zoom to Cape Town. Spatial locator map available in S2 Fig.

Maps of the percentage of microbiologically confirmed RR-tuberculosis cases at clinics revealed marked spatial heterogeneity (Fig 3). By mapping at the clinic level, we achieved a higher resolution of variability than captured by subdistricts alone (Fig 2). We removed 66 clinics (17.9%) that had fewer than 20 tuberculosis episodes during this study period to avoid skewing because of a single case with a low denominator. This resulted in 581 tuberculosis episodes (0.26% of all tuberculosis episodes) being removed from these smaller locations. Although the median percentage of RR-tuberculosis diagnosed at clinics was 4.3% for the province, we found areas with very high percentages of RR-tuberculosis (maximum 25%) and others with no diagnosed RR-tuberculosis (S1 Fig). Cape Town townships and informal settlements, the rural region of the West Coast, and areas bordering the Eastern Cape and adjacent to the metropole of George had the highest RR-tuberculosis percentages (Fig 3). Additionally, there were discrete areas with high RR-tuberculosis percentages in the Swartland area and the western part of the Cape Winelands and in Zoar, a town in the Central Karoo.

**Fig 4:**
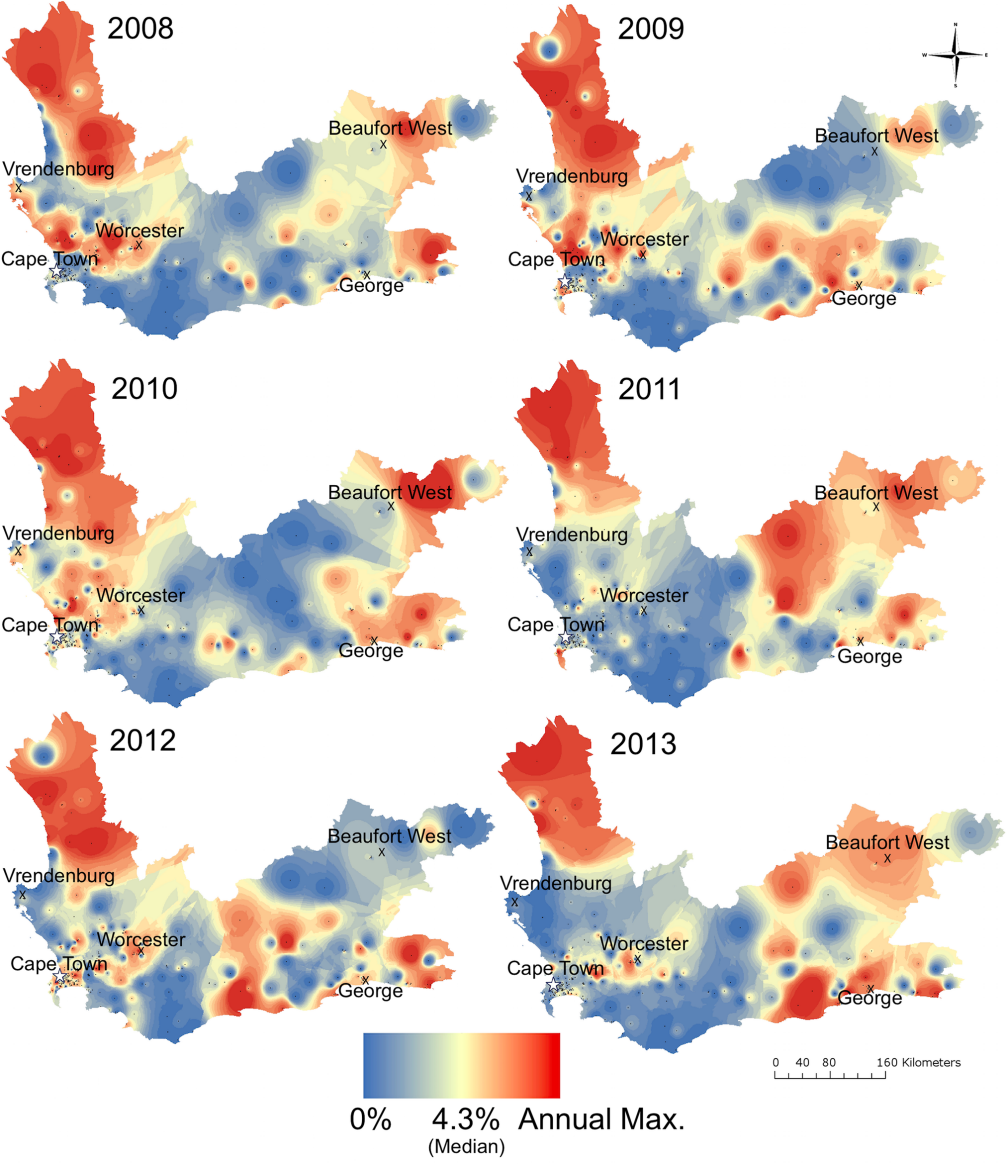
The percentage of total clinic-diagnosed tuberculosis cases found to be rifampicin resistant in Western Cape Province, yearly, for 2008–2013. Each year has an associated spectrum of interpolated percentages, beginning with the percentage of total clinic-diagnosed tuberculosis cases found to be RR-tuberculosis equal to 0 set at blue, with a median value of 4.3% (provincial average) at yellow, and the year-specific maximum (Max.) percentage in red. X’s denote large towns and the star denotes the Cape Town metropole. Max., year-specific maximum; RR, rifampicin-resistant.

**Fig 5:**
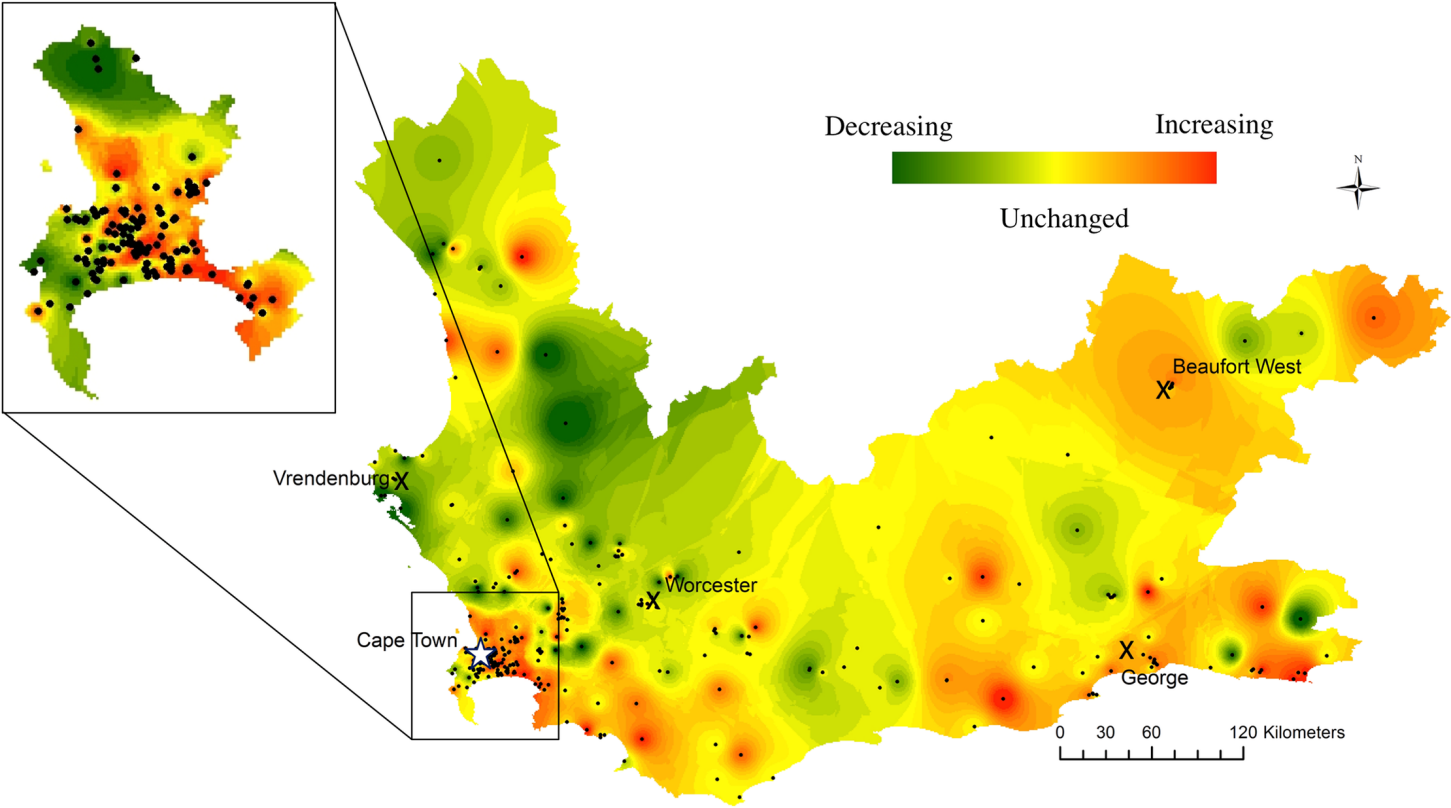
Interpolation heatmap of the statistically significant change in rifampicin resistance as a percentage of clinic-diagnosed tuberculosis during 2008–2013. Red indicates increased rifampicin resistance percentage. Green indicates decreased rifampicin resistance percentage. Yellow indicates no significant change in rifampicin resistance percentages. Intensity of color denotes degree of statistical significance. Black dots denote clinic locations, X’s denote large towns, and the star denotes the Cape Town metropole. Zoom to Cape Town.

When case counts were stratified by year, 252 clinics (83%) had stable RR-tuberculosis percentages, while 40 (13%) had increasing and 10 (3%) had decreasing percentages over time (Figs 4 and 5). For example, the West Coast initially had a very high percentage of RR-tuberculosis that decreased by 2012–2013. Conversely, communities bordering the Eastern Cape Province had higher RR-tuberculosis percentages in 2010–2013 compared to 2008–2009. The RR-tuberculosis percentage increased in many townships and informal settlement clinics in Cape Town during this time period.

## Discussion

Our data aggregation, cleaning, linkage, and mapping method detected substantial heterogeneity in the RR-tuberculosis case percentages across the Western Cape Province, with the percentage of tuberculosis cases found to be RR at clinics ranging from 0% to 25%. This wide variability in drug resistance among clinics indicates an urgent need for more granular surveillance to locate emerging and chronic hot spots. Through this project, we have developed a road map for organizing routinely collected laboratory data from the South African NHLS, showing that molecular diagnostics can be transformed into a meaningful surveillance tool.

The South African Tuberculosis Drug Resistant Tuberculosis Survey from 2012–2014 estimated that 4.3% of tuberculosis cases in the Western Cape Province were RR, a figure very close to our province-wide mean estimate of 4.6% [12]. We successfully identified areas known to have a high burden of RR-tuberculosis, such as poorer townships in Cape Town [25], communities near George [26], and an outbreak in the West Coast District (Fig 3)[27]. We also identified areas warranting further investigation for RR-tuberculosis, such as rural wine and farming regions and communities bordering the Eastern Cape Province—an area with poorer healthcare infrastructure, known higher drug-resistant tuberculosis rates, and from where drug-resistant tuberculosis cases have been linked via molecular epidemiology studies [28–30].

Our subdistrict maps reveal that high-burden tuberculosis and RR-tuberculosis regions disaggregate spatially, reflecting that high RR-tuberculosis burden does not always derive from higher tuberculosis burden (Fig 2). This disaggregation has been observed in other settings as well, leading to a consensus that tuberculosis disease burden per se is not sufficient to predict drug resistance clusters [14]. Additionally, the change in RR-tuberculosis percentages did not change uniformly nor unidirectionally across the province (Figs 4 and 5). The spatiotemporal variability of RR-tuberculosis may be driven in part by initiatives implemented during our study period, including more enrollment of HIV-coinfected individuals into antiretroviral therapy (ART) programs and the use of Xpert MTB/RIF in the diagnostic algorithm. For example, Nanoo and colleagues showed that scale-up of ART had differential impact on pulmonary tuberculosis incidence between 2004 and 2012 in different South African provinces, potentially reflecting variation in ART rollout or HIV prevalence in different regions [31]. Cox and colleagues reported that Xpert MTB/RIF rollout had a differential impact on the percentage of patients initiating MDR-tuberculosis treatment between provinces, in part dependent on successful linkage to care [32]. Our results suggest that these differential effects are identifiable at levels below the provincial scale.

To map the NHLS routinely collected laboratory results, we made critical decisions on how to organize the data. First, we chose to map RR-tuberculosis because this diagnosis should trigger initiation of an MDR regimen (second-line drugs), and current molecular techniques more accurately capture rifampicin than isoniazid; mapping only MDR-tuberculosis cases could have led to missing up to 10% of cases because of misclassification of isoniazid susceptibility [33]. Second, we aggregated at clinics rather than home addresses because the laboratory system more accurately captures clinic locations. Third, we assumed that tuberculosis is not a diagnosis for which individuals travel far for treatment except in cases of severe disease, making clinics an acceptable proxy for recent residence. Kapwata and colleagues have reported that individuals with XDR-tuberculosis traveled long distances for diagnosis in KwaZulu-Natal between 2011 and 2014 [34]. Although XDR-tuberculosis is a more severe form of the disease that might not generalize to all tuberculosis, future work should confirm whether burden of disease at a particular clinic reflects its surrounding community or is a surrogate for reputation of care quality. Fourth, we assumed similar adherence to the rifampicin drug susceptibility testing algorithm across locations. Almost half of the individuals with confirmed tuberculosis in our dataset had rifampicin susceptibility documented. This approach is supported by our finding that our percentage of RR-tuberculosis adjusted for all tuberculosis cases was similar to the drug resistance survey results of this period [12]. The percentage of RR-tuberculosis cases documented also did not change drastically after 2011 when Xpert MTB/RIF was implemented, leading to more complete DST being performed (3.9%–4.6% pre-Xpert MTB/RIF versus 4.4%–6.7% post-Xpert MTB/RIF). In reality, our maps may in part reflect clinics that variably apply the testing algorithm, meaning the variation in our maps could be driven by a combination of disease burden and facility quality.

Our approach and findings had several limitations. Our person-matching algorithm is an approximation. Others have produced estimates of province-wide tuberculosis and MDR-tuberculosis case counts from the same NHLS specimen database using their own person-linking algorithms but have not focused on those individuals diagnosed at mappable clinic settings, explaining our lower total numbers [31,35,36]. Additionally, we erred on the side of more conservative matches to avoid repeatedly counting the sample individual, but this may have led to counting two individuals as the same person. It is clear that South Africa would benefit from adoption of unique patient identifiers integrated into the specimen capture systems to support stronger surveillance and tracking [37,38].

Next, we note that the number of tuberculosis and MDR-tuberculosis cases diagnosed decreased yearly during our study period (Table 1). Because we assign tuberculosis episodes to the first year a positive result is identified, our 2008 and possibly early 2009 numbers are inflated by individuals who had treatment initiated in earlier years and would have been assigned to 2007 or earlier if we had those data. Based on the data presented in Table 1, we can estimate the number of cases in 2008 that would have been assigned to 2007, had we had 2007 data. The number of individuals who tested positive in 2009 and were excluded because of also testing positive in 2008 was 3,373, representing 7.6% of reported cases in 2009. This implies that approximately 7.5% of the cases reported in 2008 were potentially from individuals who would have tested positive in 2007 if those data were available. This would lead to an adjusted 2008 case count of 46,000 cases, suggesting a 30% decline between 2008 and 2012. A decline of 15%–20% has been reported by others, hypothesized because of changes in the diagnostic algorithm and increased access to ART [39–41]. In 2011, when Xpert MTB/RIF was rolled out, the initial predictions were that more tuberculosis cases would be identified. It is unclear whether the lower numbers reported, especially in 2012, reflect changes in testing approaches or how data were collected and aggregated in the central NHLS after the Xpert rollout. Our aggregated numbers from the first half of 2013 appear on track to be closer to 2011. We were also limited by lack of access to a complete set of Xpert results after its 2011 rollout and therefore based our estimates on smear, mycobacterial culture, and LPA results, which are the confirmatory tests. True tuberculosis cases could have been missed that were found only with a positive Xpert result; we believe this approach to aggregation and mapping would benefit from application to a dataset containing complete Xpert results, as well. A challenge with Xpert is how to interpret specimens that are positive only by its testing and subsequently without confirmation on smear or culture [42].

Third, as noted above, we omitted non-clinic locations from our analysis and mapping because of their lack of spatial relevance to an individual’s residence. Our approach could introduce bias if individuals with tuberculosis or RR-tuberculosis enter those facilities from particular locations more frequently. The South African Department of Health protocol is to have persons being evaluated for tuberculosis to do so at the clinic level and be referred to hospital only if they are too ill to receive care in the community [17]. Patients diagnosed in hospitals and lacking a clinic specimen are likely those who were most ill, supported by our finding of higher percentages of RR-tuberculosis among tuberculosis cases solely from non-clinic locations compared to clinics (6.7% versus 4.6%). An alternative possibility for the higher RR-tuberculosis percentage in the non-clinic locations (which were dominated by hospitals and prisons) is that it reflects improved adherence to the testing algorithm. This biasing, due to more severely ill patients or differences in testing, could introduce underestimation in some of our numbers, making our evaluation potentially conservative. Our data are limited to the public sector, which is where the majority of tuberculosis testing occurs in South Africa (estimated 93% in 2012–2013), but we could have underestimated case burden if particular communities access the private sector disproportionately [43]. We also assume in our mapping approach that areas between clinics reflect the percentage at the most proximal clinic, whereas there could be areas where this relationship is more complex because of population density or geography. We removed small clinics that had fewer than 20 tuberculosis episodes because a single RR-tuberculosis case could skew the percentages because of the low denominator; their removal may have impacted on capturing this complexity in shading between larger clinics.

In summary, we demonstrate a novel method to leverage routinely collected laboratory data to estimate the distribution of microbiologically confirmed RR-tuberculosis disease in a population. In the future, this framework could allow public health providers to have near real-time surveillance of drug resistance burden, evaluate programmatic interventions, and monitor progress towards national and global tuberculosis reduction goals. This approach is in contrast to the current approach of conducting large, costly, and often too infrequent drug resistance surveys, the most recent of which in South Africa, 2012–2014, was the first undertaken in 10 years.

Recent evidence from models and molecular data indicates that MDR-tuberculosis infection in high-burden settings is overwhelmingly due to transmission of preexisting MDR strains (>70%), rather than acquisition through selection of drug-resistant mutations during previous ineffective treatment, and that MDR-tuberculosis due to transmission will increase in the coming years [44–46]. Preventing MDR-tuberculosis transmission requires different control methods than preventing drug-resistant acquisition, including an increased focus on real-time knowledge of where active drug-resistant cases are clustering, in order to identify cases and initiate treatment. Maps such as the ones we have created can be the platform for this next generation of disease surveillance. The epidemic appears very dynamic, requiring constant monitoring. Improved knowledge of subnational geographic variability of RR-tuberculosis is essential for the improved design and implementation of national and local responses to reduce drug-resistant tuberculosis transmission and for timely context-specific resource allocation.

## Supporting information

S1 Checklist STROBE/RECORD checklist. (DOCX)

S1 Text Person-matching algorithm. (DOCX)

S2 Text Definition of tuberculosis episode included in our mapping. (DOCX)

S3 Text Definition of RR-tuberculosis. RR, rifampicin-resistant.
(DOCX)

S4 Text Construction of maps of spatially smoothed RR-tuberculosis percentages. RR, rifampicin-resistant.
(DOCX)

S5 Text Data cleaning processes leading up to running the person-matching algorithm. (DOCX)

S1 Table Non-clinic locations to which samples were submitted and subsequently removed from this analysis and percentage of total samples. (DOCX)

S1 Fig Distribution of RR-tuberculosis percentages by clinic over the entire study period. The median percentage is 4.3%. RR, rifampicin-resistant.
(TIF)

S2 Fig Map of the Western Cape Province, South Africa, highlighting geography referenced within the manuscript. The key describes how font variations indicate various levels of political geography.
(TIF)

## Abbreviations

AFB: acid-fast bacilli
ART: antiretroviral therapy
DST: drug susceptibility test
IDW: inverse distance weighting
LPA: Genotype MTBDR*plus* line probe assay
MDR: multidrug-resistant
NHLS: National Health Laboratory Services
RR: rifampicin-resistant
WHO: World Health Organization
Xpert: Xpert MTB/RIF
