# Supplementary material for: Using routinely collected laboratory data to identify high rifampicin-resistant tuberculosis burden communities in the Western Cape Province, South Africa: A retrospective spatiotemporal analysis
Source: PLoS Med. 2018 Aug 21;15(8):e1002638. doi: 10.1371/journal.pmed.1002638 (PMC6103505; doi:10.1371/journal.pmed.1002638)
Supplement: S1 Checklist — (DOCX) [file pmed.1002638.s001.docx]

**S1 Checklist: STROBE/RECORD Checklist** – checklist of items, extended from the STROBE statement, that should be reported in observational studies using routinely collected health data.

|  | **Item No.** | **STROBE items** | **Location in manuscript where items are reported** | **RECORD items** | **Location in manuscript where items are reported** |
| --- | --- | --- | --- | --- | --- |
| **Title and abstract** | | | | | |
|  | 1 | (a) Indicate the study’s design with a commonly used term in the title or the abstract (b) Provide in the abstract an informative and balanced summary of what was done and what was found | (a) Our title indicates our study design*.*  (b) Our abstract summarizes the study objective and key findings. | RECORD 1.1: The type of data used should be specified in the title or abstract. When possible, the name of the databases used should be included.  RECORD 1.2: If applicable, the geographic region and timeframe within which the study took place should be reported in the title or abstract.  RECORD 1.3: If linkage between databases was conducted for the study, this should be clearly stated in the title or abstract. | - 1. abstract   2. abstract   3. abstract |
| **Introduction** | | | | | |
| Background rationale | 2 | Explain the scientific background and rationale for the investigation being reported | In introduction paragraphs 1 and 2 we provide the scientific rationale for this analysis. |  |  |
| Objectives | 3 | State specific objectives, including any pre-specified hypotheses | In introduction paragraph 3 we describe our objective. |  |  |
| **Methods** | | | | | |
| Study Design | 4 | Present key elements of study design early in the paper | Methods paragraph 2 provides a description of our person-matching algorithm. Methods paragraph 4 describes assignment of geographic location. |  |  |
| Setting | 5 | Describe the setting, locations, and relevant dates, including periods of recruitment, exposure, follow-up, and data collection | Methods paragraph 1 describes the NHLS database and the population it represents. |  |  |
| Participants | 6 | *(a) Cohort study* - Give the eligibility criteria, and the sources and methods of selection of participants. Describe methods of follow-up  *Case-control study* - Give the eligibility criteria, and the sources and methods of case ascertainment and control selection. Give the rationale for the choice of cases and controls  *Cross-sectional study* - Give the eligibility criteria, and the sources and methods of selection of participants  *(b) Cohort study* - For matched studies, give matching criteria and number of exposed and unexposed  *Case-control study* - For matched studies, give matching criteria and the number of controls per case | Methods paragraphs 2-4 describe our eligibility criteria at the specimen level. We describe the circumstances for which we increased the stringency of our person-matching algorithm and how we handle when individuals had multiple positive tests. Methods paragraph 4 describes criteria at the individual level, including how individuals from non-clinic locations were handled. Lastly in the methods, paragraph 4, at the clinic level we describe eligibility at the clinic-level. | RECORD 6.1: The methods of study population selection (such as codes or algorithms used to identify subjects) should be listed in detail. If this is not possible, an explanation should be provided.  RECORD 6.2: Any validation studies of the codes or algorithms used to select the population should be referenced. If validation was conducted for this study and not published elsewhere, detailed methods and results should be provided.  RECORD 6.3: If the study involved linkage of databases, consider use of a flow diagram or other graphical display to demonstrate the data linkage process, including the number of individuals with linked data at each stage. | 6.1) The person-matching algorithm is described in detail in S1 Text.  6.2) N/A  6.3) N/A |
| Variables | 7 | Clearly define all outcomes, exposures, predictors, potential confounders, and effect modifiers. Give diagnostic criteria, if applicable. | In methods paragraph 3, we defined a case (i.e., the outcome) of microbiologically-confirmed tuberculosis. Both definitions are further detailed in S2 & S3 Text. | RECORD 7.1: A complete list of codes and algorithms used to classify exposures, outcomes, confounders, and effect modifiers should be provided. If these cannot be reported, an explanation should be provided. | In methods paragraph 3, we defined a case (i.e., the outcome) of microbiologically-confirmed tuberculosis. Both definitions are further detailed in S2 & S3 Text. |
| Data sources/ measurement | 8 | For each variable of interest, give sources of data and details of methods of assessment (measurement).  Describe comparability of assessment methods if there is more than one group | All data for this cohort is obtained from the NHLS database. The methods used to assess microbiologically-confirmed TB and RR-tuberculosis were the same across all participants. |  |  |
| Bias | 9 | Describe any efforts to address potential sources of bias | We address how we handled small clinics in STROBE question 6. |  |  |
| Study size | 10 | Explain how the study size was arrived at | Our sample size was determined by the availability of data, with the process described in methods paragraphs 2-4. |  |  |
| Quantitative variables | 11 | Explain how quantitative variables were handled in the analyses. If applicable, describe which groupings were chosen, and why | The methods used to handle quantitative variables are described in methods paragraphs 2-4. |  |  |
| Statistical methods | 12 | (a) Describe all statistical methods, including those used to control for confounding  (b) Describe any methods used to examine subgroups and interactions  (c) Explain how missing data were addressed  (d) *Cohort study* - If applicable, explain how loss to follow-up was addressed  *Case-control study* - If applicable, explain how matching of cases and controls was addressed  *Cross-sectional study* - If applicable, describe analytical methods taking account of sampling strategy  (e) Describe any sensitivity analyses | (a) Our statistical and geospatial mapping methods are described in methods paragraphs 6-8, as well as in further detail in S4 Text. We describe how we used  (b) Subgroup analyses are detailed in methods paragraphs 6-8.  (c) The handling of missing data is described in S1 Text.  (d) & (e) N/A |  |  |
| Data access and cleaning methods |  | .. |  | RECORD 12.1: Authors should describe the extent to which the investigators had access to the database population used to create the study population.  RECORD 12.2: Authors should provide information on the data cleaning methods used in the study. | 12.1) In the methods paragraph 1, we describe the completeness of the available data.  12.2) We provide details of data cleaning methods in S5 Text. |
| Linkage |  | .. |  | RECORD 12.3: State whether the study included person-level, institutional-level, or other data linkage across two or more databases. The methods of linkage and methods of linkage quality evaluation should be provided. | Methods paragraphs 2-4 detail how the specimen-level database was used to develop a person-level database. The methodology used and the person-matching algorithm are described in detail in S1 Text. |
| **Results** | | | | | |
| Participants | 13 | (a) Report the numbers of individuals at each stage of the study (*e.g.*, numbers potentially eligible, examined for eligibility, confirmed eligible, included in the study, completing follow-up, and analysed)  (b) Give reasons for non-participation at each stage.  (c) Consider use of a flow diagram | (a) Results paragraphs 1-2 report the number of individuals at each stage of the study. This is visually represented in the Fig 1 flow diagram.  (b) Reasons for exclusion are outlined in STROBE question 6a.  (c) We present a flow diagram in Fig 1. | RECORD 13.1: Describe in detail the selection of the persons included in the study (*i.e.,* study population selection) including filtering based on data quality, data availability and linkage. The selection of included persons can be described in the text and/or by means of the study flow diagram. | The selection of the study population is detailed in methods paragraph 4. |
| Descriptive data | 14 | (a) Give characteristics of study participants (*e.g.*, demographic, clinical, social) and information on exposures and potential confounders  (b) Indicate the number of participants with missing data for each variable of interest  (c) *Cohort study* - summarise follow-up time (*e.g.*, average and total amount) | (a) Because this cohort is built from a laboratory database, demographic variables are limited. In results paragraph 2, we report basic demographics.  (b) See our response to STROBE question 12c and S1 Text.  (c) Although this analysis was primarily cross-sectional, in results paragraph 2 we report that percent of individuals with confirmed TB had more than one episode of disease during the study period. How these individuals were handled is detailed further in S2 Text. |  |  |
| Outcome data | 15 | *Cohort study* - Report numbers of outcome events or summary measures over time  *Case-control study* - Report numbers in each exposure category, or summary measures of exposure  *Cross-sectional study* - Report numbers of outcome events or summary measures | The summary measure is reported in results paragraph 4 (also see spatial representation in Fig 2 and 3). The outcome measure over time (i.e., per year) is reported in Table 1 and spatially in Figs 4 and 5. |  |  |
| Main results | 16 | (a) Give unadjusted estimates and, if applicable, confounder-adjusted estimates and their precision (e.g., 95% confidence interval). Make clear which confounders were adjusted for and why they were included  (b) Report category boundaries when continuous variables were categorized  (c) If relevant, consider translating estimates of relative risk into absolute risk for a meaningful time period | (a) We provide estimates in results paragraph 1.  (b) & (c) N/A |  |  |
| Other analyses | 17 | Report other analyses done—e.g., analyses of subgroups and interactions, and sensitivity analyses | In addition to looking at the percentages of RR-tuberculosis across the province over the years, we also report in Fig 2 the tuberculosis case counts and RR-tuberculosis case counts per subdistrict, both as absolute numbers and adjusted for population. We also look at the trend of RR-tuberculosis percentage over the five and a half year study period, depicting statistically significant trends in Fig 5. |  |  |
| **Discussion** | | | | | |
| Key results | 18 | Summarise key results with reference to study objectives | In our discussion we highlight that our key findings, particularly in discussion paragraphs 1-3. |  |  |
| Limitations | 19 | Discuss limitations of the study, taking into account sources of potential bias or imprecision. Discuss both direction and magnitude of any potential bias | In discussion paragraphs 5-7, we outline the limitations of our analysis. | RECORD 19.1: Discuss the implications of using data that were not created or collected to answer the specific research question(s). Include discussion of misclassification bias, unmeasured confounding, missing data, and changing eligibility over time, as they pertain to the study being reported. | In discussion paragraphs 5-7, we outline the limitations of our analysis. |
| Interpretation | 20 | Give a cautious overall interpretation of results considering objectives, limitations, multiplicity of analyses, results from similar studies, and other relevant evidence | In discussion paragraph 2, we compare our findings to the 2012-2014 South African Tuberculosis Drug Resistant Tuberculosis Survey. |  |  |
| Generalisability | 21 | Discuss the generalisability (external validity) of the study results | See response to STROBE question 20. |  |  |
| **Other Information** | | | | | |
| Funding | 22 | Give the source of funding and the role of the funders for the present study and, if applicable, for the original study on which the present article is based | See the funding information section. |  |  |
| Accessibility of protocol, raw data, and programming code |  | .. |  | RECORD 22.1: Authors should provide information on how to access any supplemental information such as the study protocol, raw data, or programming code. | See the Data Availability Statement. |

*Reference: Benchimol EI, Smeeth L, Guttmann A, Harron K, Moher D, Petersen I, Sørensen HT, von Elm E, Langan SM, the RECORD Working Committee. The REporting of studies Conducted using Observational Routinely-collected health Data (RECORD) Statement. *PLoS Medicine* 2015; in press.

*Checklist is protected under Creative Commons Attribution ([CC BY](http://creativecommons.org/licenses/by/4.0/)) license.
