# Supplementary material for: Using routinely collected laboratory data to identify high rifampicin-resistant tuberculosis burden communities in the Western Cape Province, South Africa: A retrospective spatiotemporal analysis
Source: PLoS Med. 2018 Aug 21;15(8):e1002638. doi: 10.1371/journal.pmed.1002638 (PMC6103505; doi:10.1371/journal.pmed.1002638)
Supplement: S1 Text — (DOCX) [file pmed.1002638.s002.docx]

**S1 Text: Person-Matching Algorithm:**

We first applied a less strict algorithm to create unique identifiers as follows: (a) after cleaning, which removed 161,054 specimens (4%) due to missing data, the entire dataset was sorted by a combination of last name and first four initials of first names (name block), (b) within each name block, specimens that had the same age or were within 1 year, AND (had same sex OR same location) were designated as the same episode and given a numeric indicator, 1,2,3, etc. For specimens linked to common names, defined as those having >20 laboratory entries in the dataset (since more than 20 samples from the same person is highly unlikely to have occurred from the same disease episode), which is <1% of all samples, we used a stricter algorithm. This stricter procedure changes the OR between same sex and same location to an AND, enforcing matching on both sex and location rather than one or the other, therefore removing flexibility if one of those entries had an entry error. The stricter algorithm would classify the same person as two persons if that individual moved between locations. However, this restriction was only applied to <1% of samples and was needed to disaggregate common names.
