# Supplementary material for: Using routinely collected laboratory data to identify high rifampicin-resistant tuberculosis burden communities in the Western Cape Province, South Africa: A retrospective spatiotemporal analysis
Source: PLoS Med. 2018 Aug 21;15(8):e1002638. doi: 10.1371/journal.pmed.1002638 (PMC6103505; doi:10.1371/journal.pmed.1002638)
Supplement: S2 Text — (DOCX) [file pmed.1002638.s003.docx]

**S2 Text: Definition of tuberculosis episode included in our mapping**

A tuberculosis episode is defined as the following:

1. An individual has a clinic location for a submitted specimen during the study period and is assigned to that clinic location. If an individual moves between locations, that individual is assigned to the first clinic location where microbiologic confirmation of tuberculosis is made, or the clinic closest in time if the diagnosis is made at a nonclinic location.
2. An individual has a specimen with microbiologic confirmation of tuberculosis (a positive AFB smear and/or mycobacterial culture result)
3. An individual is without evidence of a microbiologically confirmed tuberculosis episode in the previous year. For instance, if an individual has their first positive tuberculosis test in 2009, they are not eligible to have a new tuberculosis episode until 2011. Any positive tests in 2009 and 2010 will be considered part of the tuberculosis episode assigned to 2009 since a repeat positive sample most likely reflects not yet responding to therapy, treatment failure, or relapse rather than a discrete new disease episode.
