# Supplementary material for: Using routinely collected laboratory data to identify high rifampicin-resistant tuberculosis burden communities in the Western Cape Province, South Africa: A retrospective spatiotemporal analysis
Source: PLoS Med. 2018 Aug 21;15(8):e1002638. doi: 10.1371/journal.pmed.1002638 (PMC6103505; doi:10.1371/journal.pmed.1002638)
Supplement: S3 Text — RR, rifampicin-resistant. (DOCX) [file pmed.1002638.s004.docx]

**S3 Text: Definition of rifampicin resistant tuberculosis**

To determine rifampicin resistance, we use the following rules:

1. Rifampicin resistance is determined using MTBDR*plus* or DST on culture. These are the confirmatory tests for rifampicin resistance so they were used as determinants (rather than Xpert MTB/RIF).
2. If an individual tests rifampicin resistant (RR) anytime during their tuberculosis episode, then that episode is a RR-tuberculosis episode. For instance, if an individual tests positive for tuberculosis in 2009 and then separately tests positive for RR in 2010, that tuberculosis episode is labeled as one RR-tuberculosis episode. If that same individual again tests positive for TB in 2012, RR would be determined by samples taken in 2012 and 2013 for that second TB episode.
