# Supplementary material for: Using routinely collected laboratory data to identify high rifampicin-resistant tuberculosis burden communities in the Western Cape Province, South Africa: A retrospective spatiotemporal analysis
Source: PLoS Med. 2018 Aug 21;15(8):e1002638. doi: 10.1371/journal.pmed.1002638 (PMC6103505; doi:10.1371/journal.pmed.1002638)
Supplement: S4 Text — RR, rifampicin-resistant. (DOCX) [file pmed.1002638.s005.docx]

**S4 Text: Construction of maps of spatially smoothed rifampicin resistant tuberculosis percentages**

Case counts of tuberculosis and rifampicin resistant (RR) tuberculosis were aggregated at the clinic level for each year and over the entire study period. We used a standard inverse distance weighting (IDW) technique implemented with ArcGIS version 10.3 to pixelate the Western Cape and generate a color-coded interpolated RR-tuberculosis percentage, defined as the percentage of total tuberculosis cases that were RR-tuberculosis, for each unobserved pixel. The interpolated value is a weighted average of known neighboring clinic RR-tuberculosis percentages, with a penalty for distance from the clinics informing the weighted average. Each pixel value *u* at location *x* is a function of the RR-tuberculosis percentages found at clinics surrounding it. The formula for interpolated value *u* at location *x* is:

$u(x)=\sum_{i=0}^{N} (\frac{w_{i}(x)u_{i}}{\sum_{j=0}^{N} w_{j}(x)})$, with $w_{i}(x)=\frac{1}{d(x,x_{i})^{p}}$,

where *u_i_* denotes the observed RR-tuberculosis percentage at location *x_i_*, function *d* denotes the Euclidean distance between *x* and *x_i_*, *N* denotes the total number of observed locations, and *p* is a positive real number; we use the default ArcGIS IDW setting of 2 for *p*.
