# Supplementary material for: Using routinely collected laboratory data to identify high rifampicin-resistant tuberculosis burden communities in the Western Cape Province, South Africa: A retrospective spatiotemporal analysis
Source: PLoS Med. 2018 Aug 21;15(8):e1002638. doi: 10.1371/journal.pmed.1002638 (PMC6103505; doi:10.1371/journal.pmed.1002638)
Supplement: S5 Text — (DOCX) [file pmed.1002638.s006.docx]

**S5 Text: Data cleaning processes leading up to running the person-matching algorithm**

The final dataset used in this analysis was a product of the aggregation of five smaller datasets that were merged by unique specimen ID. Merging numerous datasets was necessitated by the fact that data extraction from the repository was limited by the number of variables that could be extracted at a given time and that updates for more recent years were acquired as the project progressed. Investigators worked with NHLS colleagues to interpret and standardize response sets to given variables, which varied by data pull, across the contributing datasets for the final dataset.
