# Supplementary material for: Using routinely collected laboratory data to identify high rifampicin-resistant tuberculosis burden communities in the Western Cape Province, South Africa: A retrospective spatiotemporal analysis
Source: PLoS Med. 2018 Aug 21;15(8):e1002638. doi: 10.1371/journal.pmed.1002638 (PMC6103505; doi:10.1371/journal.pmed.1002638)
Supplement: S1 Table — (DOCX) [file pmed.1002638.s007.docx]

**S1 Table: Non-clinic locations from which samples were submitted and subsequently removed from this analysis and percentage of total samples**

| Location type | Number of Samples | Percentage of total Samples |
| --- | --- | --- |
| Regional/District Hospital | 201,827 | 9.1 |
| Correctional Service | 50,801 | 2.2 |
| Tuberculosis Hospital | 47,565 | 2.1 |
| Secondary Hospital | 44,683 | 2.0 |
| Specialized Psychiatric Facility | 5,186 | 0.23 |
| Hospice | 1,764 | 0.08 |
| Military Facility | 1,113 | 0.05 |
| Rehabilitation Centre | 328 | <0.01 |
| Women’s Shelter | 294 | <0.01 |
| Private Hospital | 217 | <0.01 |
