# Supplementary figures and images for: Using routinely collected laboratory data to identify high rifampicin-resistant tuberculosis burden communities in the Western Cape Province, South Africa: A retrospective spatiotemporal analysis
Source: PLoS Med. 2018 Aug 21;15(8):e1002638. doi: 10.1371/journal.pmed.1002638 (PMC6103505; doi:10.1371/journal.pmed.1002638)

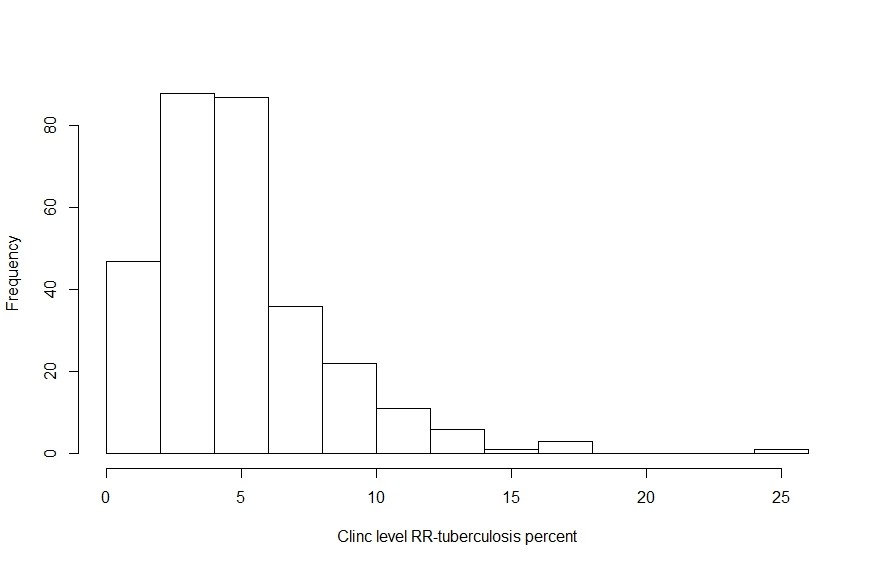

Supplement: S1 Fig — The median percentage is 4.3%. RR, rifampicin-resistant. (TIF) [file pmed.1002638.s008.tif]

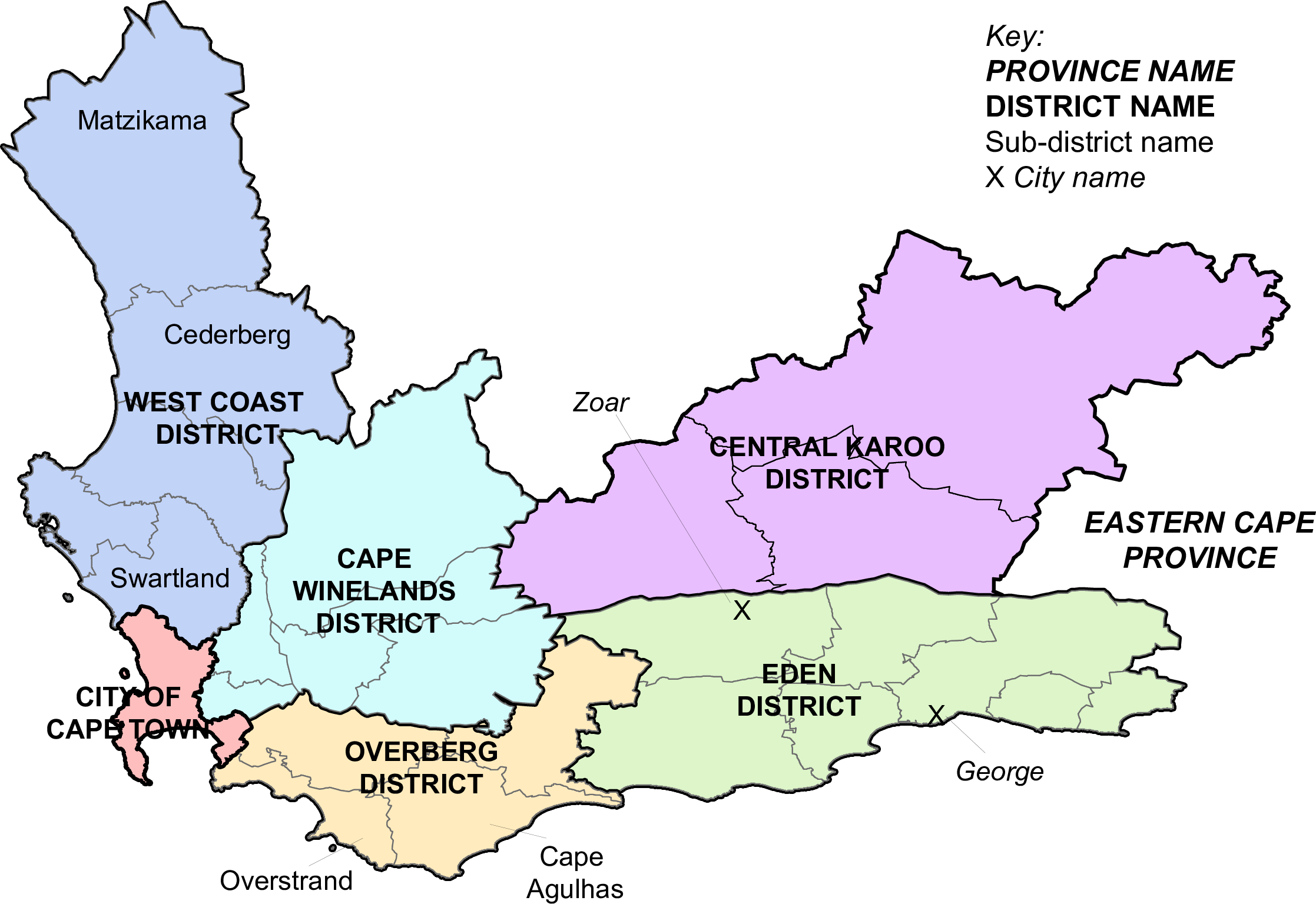

Supplement: S2 Fig — The key describes how font variations indicate various levels of political geography. (TIF) [file pmed.1002638.s009.tif]
